# Laquinimod attenuates inflammation by modulating macrophage functions in traumatic brain injury mouse model

**DOI:** 10.1186/s12974-018-1075-y

**Published:** 2018-01-30

**Authors:** Atsuko Katsumoto, Aline S. Miranda, Oleg Butovsky, Antônio L. Teixeira, Richard M. Ransohoff, Bruce T. Lamb

**Affiliations:** 1 Department of Neurosciences, Lerner Research Institute, Cleveland Clinic Foundation, Cleveland, OH USA; 2 Stark Neurosciences Research Institute, Indiana University School of Medicine, 320 W 15th St, Indianapolis, IN 46202 USA; 3 Laboratory of Neurobiology, Department of Morphology, Federal University of Minas Gerais, Belo Horizonte, MG Brazil; 4 Center of Neurologic Diseases, Brigham and Women’s Hospital, Boston, MA USA; 5 Interdisciplinary Laboratory of Medical Investigation, School of Medicine, Federal University of Minas Gerais, Belo Horizonte, MG Brazil

**Keywords:** Laquinimod, Microglia, Peripherally derived monocytes, Traumatic brain injury

## Abstract

**Background:**

Traumatic brain injury (TBI) is a critical public health and socio-economic problem worldwide. A growing body of evidence supports the involvement of inflammatory events in TBI. It has been reported that resident microglia and infiltrating monocytes promote an inflammatory reaction that leads to neuronal death and eventually behavioral and cognitive impairment. Currently, there is no effective treatment for TBI and the development of new therapeutic strategies is a scientific goal of highest priority. Laquinimod, an orally administered neuroimmunomodulator initially developed for the treatment of multiple sclerosis, might be a promising neuroprotective therapy for TBI. Herein, we aim to investigate the hypothesis that laquinimod will reduce the central nervous system (CNS) damage caused by TBI.

**Methods:**

To test our hypothesis, *Ccr2*^rfp/+^ *Cx3cr1*^*gfp/+*^ mice were submitted to a moderate TBI induced by fluid percussion. Sham controls were submitted only to craniotomy. Mice were treated daily by oral gavage with laquinimod (25 mg/kg) 7 days before and 3 days after TBI. The brains of mice treated or not treated with laquinimod were collected at 3 and 120 days post injury, and brain morphological changes, axonal injury, and neurogenesis were evaluated by microscopy analysis. We also isolated microglia from infiltrating monocytes, and the expression of immune gene mRNAs were analyzed by employing a quantitative NanoString nCounter technique.

**Results:**

Laquinimod prevented ventricle enlargement caused by TBI in the long term. Immunohistochemical analyses revealed decreased axonal damage and restored neurogenesis in the laquinimod-treated TBI group at early stage (3 days post injury). Notably, laquinimod inhibited the monocytes infiltration to the brain. Hierarchial clustering demonstrated that the microglial gene expression from the TBI group treated with laquinimod resembles the sham group more than the TBI-water control group.

**Conclusions:**

Administration of laquinimod reduced lesion volume and axonal damage and restored neurogenesis after TBI. Laquinimod might be a potential therapy strategy to improve TBI long-term prognosis.

**Electronic supplementary material:**

The online version of this article (10.1186/s12974-018-1075-y) contains supplementary material, which is available to authorized users.

## Background

Traumatic brain injury (TBI) is a critical public health and socio-economic problem worldwide. It is the leading cause of death and disability among children and young adults, although the incidence in the elderly population has also been rising [1]. Data from the Centers for Disease Control and Prevention (CDC) indicate that each year in the USA, 1.7 million people sustain a TBI, with 1.4 million of these injured individuals treated in emergency departments, with around 275,000 hospitalizations and 52,000 fatalities [2]. It is worth mentioning that neurological, behavioral, and cognitive deficits are well-known sequelae of TBI, which lead to long-term functional impairment and decrease in quality of life [3].

Multiple inflammatory responses follow TBI. Damage-associated molecular patterns (DAMPs) activate microglia and resident mononuclear phagocytes in the central nervous system (CNS), and microglial activation leads to further neuronal damage through secretion of inflammatory cytokines and reactive specimens among other mechanisms [4, 5]. These responses may determine whether microglial cell activity leads to the clearance of tissue debris and subsequent resolution of the inflammatory response or leads to chronic inflammation. In addition to microglial activation, peripherally derived macrophages and perivascular macrophages also participate in the inflammatory response. As a result of passage time or environmental factors, microglia and/or peripherally derived monocytes and macrophages may acquire an anti-inflammatory phenotype, which is associated with the increased secretion of neurotrophic factors and enhanced phagocytic activity, which cause them to remove debris and promote regeneration [5]. At present, however, how microglia and/or monocytes become chronically activated with exaggerated and consequently neurotoxic inflammatory response is poorly understood. Therefore, it is important to reveal the molecular mechanisms regulating deleterious microglial activation and to regulate the microglial and/or monocyte response to halt progressive neuronal damage.

Laquinimod is an immunomodulatory oral drug developed for the treatment of multiple sclerosis (MS) [6–8] and has been studied in clinical trials for other diseases including Huntington’s disease [9], Crohn’s disease [10], and lupus nephritis [11]. Laquinimod inhibited the infiltration of inflammatory monocytes into the CNS in experimental autoimmune encephalomyelitis (EAE), an animal model of MS [12]. In addition, CCR2 and CCL2 levels, a chemokine signaling known to be crucial for monocyte chemotaxis, were not elevated in the spinal cord of laquinimod-treated EAE mice [12]. In cultured human monocytes, laquinimod inhibited the phosphorylation state of the inflammatory signaling pathways p38/MAPK and JNK. On the other hand, the activation of human microglia by lipopolysaccharide (LPS) increased the levels of several pro- and anti-inflammatory cytokines, including tumor necrosis factor (TNF) and interleukin (IL)-6 and IL-10, which were attenuated by laquinimod [13]*.* Laquinimod also inhibited LPS-elevated phosphorylation of JNK, AKT, and 90RSK, but not of ERK1/2 and p38MAPK in human microglia [13]. Taken together, these in vivo and in vitro studies provided evidence that laquinimod modulates inflammation and may exert its effects by specifically influencing microglia and infiltrating monocyte functions.

In this scenario, we aim to investigate the effects of laquinimod treatment for lateral fluid percussion TBI in adult mice, by modifying microglia and infiltrating monocytes functions.

## Methods

### Mice

*Ccr2*^*rfp/+*^ *Cx3cr1*^*gfp/+*^ mice in which monocyte-derived macrophages and microglia are labeled with red fluorescent protein (RFP) and green fluorescent protein (GFP), respectively [14] were generated by crossbreeding *Ccr2*^rfp/rfp^::C57BL/6 mice (Jackson laboratory) [14] with *Cx3cr1*^gfp/gfp^::C57BL/6 mice (Jackson laboratory) [15]. Animals were housed at the Cleveland Clinic Biological Resources Unit, a facility fully accredited by the Association of Assessment and Accreditation of Laboratory Animal Care. All procedures were approved by the Institutional Animal Care and Use Committee of the Cleveland Clinic.

### Laquinimod administration

Laquinimod (LAQ; originally ABR-215062) was synthesized at TEVA Pharmaceutical Industries, Ltd. The compound was dissolved in purified water (25 mg/kg) and administered orally every day by oral gavage in a volume of 100 μL. LAQ was given for 7 days before the TBI for preventive treatment and for 3 days after the TBI for therapeutic treatment. The solution was stored at 4 °C and used within 1 week of preparation. Control mice received a volume of 100 μL of water by oral gavage. Each group contained between 10 and 15 animals.

### Traumatic brain injury

Lateral fluid percussion injury (LFPI) was induced in 8-week-old mice as described before [16]. Briefly, mice were anesthetized with ketamine (35.7 mg/mL)/xylazine (2.6 mg/mL), and positioning on a stereotactic frame, a craniotomy (3 mm in diameter) was made between the midline, bregma, and lambda. A plastic hub was attached around the craniotomy. After 24 h of the craniotomy, mice were anesthetized with ketamine and xylazine, and the LFPI was produced at 1.0 atm, resulting in a moderate, focal injury with a distinct cortical cavity. The skin incision was sutured. Sham controls received craniotomy, and the same amount of anesthetics without the trauma procedure.

### Histology

Mice were euthanized 3 days or 120 days after the TBI. All mice were deeply anesthetized with ketamine and xylazine and perfused transcardially with PBS, followed by PBS containing 4% paraformaldehyde. After perfusion, the brains were post-fixed in a solution of 4% paraformaldehyde at 4 °C for 24 h. Free-floating sections of the brain were prepared as previously described [17]. For immunofluorescence assay, sections were blocked with 10% normal serum for 1 h and stained with primary antibodies at 4 °C overnight. After washing with PBST (PBS with 0.1% Triton X-100) three times, the sections were incubated with secondary antibodies at room temperature for 2 h and mounted in ProLong Gold antifade reagent with DAPI (Life technologies) or in hardset FluorSave Reagent (Calbiochem). Antibodies used include mouse anti-GFP (UCDavis/NIH NeuroMab Facility, 1:8000), rabbit anti-RFP (Abcam, 1:1000), Alexa Fluor 488 goat anti-mouse IgG (Invitrogen, 1:1000), and Alexa Fluor 594 goat anti-rabbit IgG (Invitrogen, 1:1000).

Axonal pathology was assessed with amyloid precursor protein (APP) staining, and neurogenesis was assessed with doublecortin (DCX) staining. The sections were incubated in 0.3% H2O2 for 30 min at room temperature (RT) to inactivate endogenous peroxidases. Antigen retrieval was performed in × 1 target retrieval solution (Dako Cytomation) containing 0.5% Tween in PBS at 85 °C for 10 min. After blocking in 10% normal goat serum (NGS), primary rabbit anti-APP antibody (Invitrogen, 1:1000) or rabbit anti-DCX antibody (Cell signaling, 1:250) was applied overnight at 4 °C and secondary biotinylated goat anti-rabbit antibody (Vector, 1:200 for APP, 1:1000 for DCX) was applied for 1 h at RT. The signal was detected using the ABC Elite Kit (Vector) and DAB substrate kit (Vector) according to the manufacturer’s instructions. Sections were allowed to dry and were coverslipped with hardset mounting medium (Fisher Scientific). Three sections were stained for each animal and averaged after quantification.

For cresyl violet staining, brain sections were washed three times in 0.1% PBST for 10 min then mounted on glass slides. The slides were dried and immersed in xylene and graded ethanol, and de-ionized water for 3 min each. The slides were stained for 8 min in filtered cresyl violet solution, and then briefly rinsed in de-ionized water. The slides were dehydrated again in graded ethanol for 2 min each. The slices were placed in xylene for another 5 min and coverslipped.

### Measurement of brain atrophy

Brain sections were subjected to cresyl violet staining for histologic assessment of damage and the adjacent sections were processed for immunohistochemistry. The brain slices were photographed by Leica SCN400F microscope, and lesion volumes were measured by an investigator masked to the study groups with ImageJ software (National Institutes of Health).

### Imaging

Sections stained with GFP and RFP as previously described [18]. Briefly, slides were imaged on a Leica CTR5500 microscope equipped with a QImaging Retiga EXi FAST 13941 MONO camera at × 10 magnification. Tiling of consecutive fields of view was employed to capture the whole section and all sections from a mouse using ImagePro Plus software to drive the microscope. Both GFP (microglia) and RFP (monocyte) channels were recorded.

### Cell isolation and flow cytometry

Mice were deeply anesthetized with ketamine and xylazine and perfused with phosphate-buffered saline (PBS) transcardially at 3 days after the TBI. After the brains were minced and dissociated by an enzyme (Myltenyl Biotec), single mononuclear cells were isolated using a Percoll density gradient. Cells were subsequently stained with anti-CD45-Qdot605NC (eBioscience) and anti-Ly6C-Pecy7 (Biolegend). Microglia were CD45^low^ CX3CR1^high^ CCR2^neg^ and monocytes were CD45^high^ CX3CR1^low-neg^ CCR2^+^. Infiltrated monocytes were defined as CD45^high^ CX3CR1^low-neg^ CCR2^+^ Ly6C^high^ cells. Stained cells were analyzed on a LSR-II (BD Biosciences, San Jose, CA) or sorted on a FACSAria II (BD Biosciences, San Jose, CA) running Diva6. Data were analyzed with FlowJo 9 software (Treestar, Ashland, OR).

### MG468 chip design

The MG468 chip was designed using the quantitative NanoString nCounter platform [19]. Selection of genes is based on analyses that identified genes and proteins which are specifically or highly expressed in adult mouse microglia plus 40 inflammation-related genes which were significantly affected in EAE, APP-PS1, and SOD1 mice (MG400, [19]). MG468 contains additional 48 inflammation- and phagocytosis-related genes [20].

### Gene expression

Mononuclear cells were prepared from brains as previously described [21, 22]. Cells were sorted on a BD FACSAria II by gating on CD45^low^ GFP^high^ for microglia and CD45^high^ RFP^+^ for monocytes. RNA was isolated from FACS-sorted cells mixed from 5 to 13 mice from six separate experiments per group in TRIzol Reagent (Ambion) according to the manufacturer’s protocol. RNA samples were analyzed by nCounter gene expression analysis and quantified with the nCounter Digital Analyzer (NanoString Technologies). Expressions of 468 genes were analyzed using nCounter GX Mouse Inflammation kit. To minimize technical (non-biological) variability among arrays, densitometry values between arrays were normalized using the Robust Multichip Average function and further transformed to the logarithmic scale (log_2_). Gene expression levels in each sample were normalized against the geometric mean of six housekeeping genes including *Cltc*, *Gapdh*, *Gusb*, *Hprt1*, *Pgk1*, and *Tubb5*. A cutoff was introduced at the value of the highest negative control present on the chip. Fold changes were calculated using the average of each group. For each experiment, the fold changes were calculated comparing the experimental group to their appropriate controls. Based on the normalized gene expression levels of NanoString-based chips, a two tailed Student’s *t* test assuming equal variance was applied to each gene to compare the difference between the TBI group and the control group. Fold change cutoffs of > 1.5 were used to evaluate gene expression changes with number.

### Hierarchical cluster analysis

Hierarchical cluster analysis was performed using Multiple Experiment Viewer (MeV) software to see how data aggregate, and a heat map was generated with pluripotency genes.

### Real-time PCR

Total RNA was extracted using RNA clean and concentration kit (Epigenetics) according to the manufacturer’s protocol. Total RNA (50 ng/μl) was used in reverse transcription reaction (Applied Biosystems) and 3 ng RNA in 5 μL reverse transcription reaction with specific RNA probes (Applied Biosystems). qPCR reactions were performed in duplicates. Primers and probes for IL − 6 (Taqman Gene Expression Assay ID Mm00446190) and iNOS (Mm00440502) were purchased from Applied Biosystems. mRNA levels were normalized relative to GAPDH (Applied Biosystems, 4351309), by the formula 2^(−ΔCt), where ΔCt = CtmiR-X-CtGAPDH. All data are means of duplicates and the standard errors of mean were calculated between duplicates. Real-time PCR reactions were performed using Vii7 (Applied Biosystems). All qRT-PCRs were performed in duplicate, and data are presented as means ± standard errors of mean (s.e.m).

### Statistical analysis

Unless otherwise indicated, data are presented as mean ± SEM from at least three independent experiments, and *P* < 0.05 was considered statistically significant. Statistical analysis of the data was concluded using a non-parametric test (Man-Whitney or Kruskal-Wallis tests) followed by a Dunn’s multiple comparison test. All data were analyzed using GraphPad PRISM 5 software (Sandiego, CA).

## Results

### Laquinimod treatment attenuated the lateral ventricle volume following TBI

**Fig. 1:**
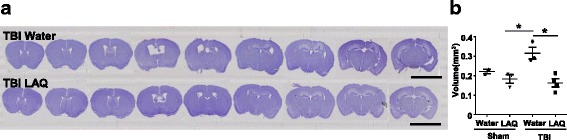
Laquinimod prevents traumatic brain injury (TBI)-induced long-term lateral ventricle enlargement. **a** Representative × 10 images of brain sections, stained with cresyl violet, from sham and TBI mice (120 days following the brain injury) treated with laquinimod (LAQ) or vehicle (water). **b** A significant increase in lateral ventricle volume was found in TBI-water group, which was significantly prevented by laquinimod treatment. Symbols denote significant differences between groups. Error bars indicate mean ± s.e.m. (*n* = 3–4/group). *p < 0.05

TBI is known to cause permanent structural and functional changes in the brain and increase the risk for neuronal loss and brain atrophy which consequently result in dementia [23, 24]. We first evaluated the effect of laquinimod in the long-term outcome of TBI. Images obtained from lateral ventricles slices stained with cresyl violet of TBI mice 120 days following the traumatic event revealed a significant enlargement of the lateral ventricles compared to sham animals (*p < 0.05) (Fig. 1a). Although there was no statistical difference in hippocampal volume (data not shown), hippocampal deformation on the ipsilateral side of the TBI-water group was not observed in the TBI-laquinimod group. Importantly, laquinimod treatment significantly prevented long-term increase in lateral ventricles volume caused by TBI (*p < 0.05) (Fig. 1b).

### Laquinimod reduced axonal damage and restored hippocampal neurogenesis followed by TBI

**Fig. 2:**
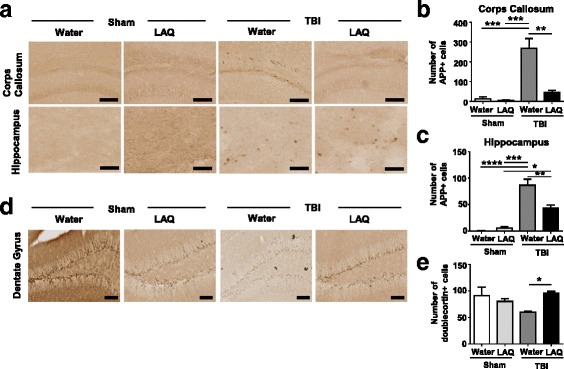
Laquinimod has neuroprotective role on TBI. **a** Representative × 20 images of APP staining in the corpus callosum and hippocampus on ipsilateral side of injury (or craniotomy for sham) from each group. **b–c** Quantification of the APP-positive cells reveals tendency of decreased axonal damage both in corpus callosum and in hippocampus in the TBI-laquinimod group compared to the TBI-water group (*n* = 3 mice for each sham group, *n* = 4 mice for each TBI group). **d** Representative × 20 images of doublecortin staining in the dentate gyrus on ipsilateral side of injury (or craniotomy for sham) from each group. **e** Quantification of neurogenesis in dentate gyrus reveals a significant increase in TBI-laquinimod group compared to the TBI-water group. Symbols denote significant differences between groups. Error bars indicate mean ± s.e.m. (*n* = 5 mice for each group). *p < 0.05, **p < 0.01, ***p < 0.001

Next, we addressed the neuropathological features at acute phase (3 days post injury). Diffuse axonal injury is one of the most common types of brain injury following TBI and independently contributes to significant morbidity and mortality. Using immunohistochemistry technique, we evaluated axonal damage in the lesion ipsilateral corpus callosum and hippocampus. Consistent with previous studies [18, 25], there was an increase in APP-positive profiles in the body of the corpus callosum as well as in hippocampus in the TBI-water group (Fig. 2a). Laquinimod treatment significantly prevented axonal damage in both areas (Fig. 2a–c). Furthermore, in the immunohistochemistry for the neuronal migration protein, doublecortin (DCX) revealed reduced hippocampal neurogenesis in dentate gyrus in the TBI-water group, which was restored to the levels observed in sham controls in TBI-laquinimod group (*p < 0.05) (Fig. 2d, e).

### Monocytes infiltration following TBI was reduced by laquinimod

CCR2 is an important chemokine involved in the recruitment of monocytes to damaged tissues in an inflammatory milieu. Axonal injury correlates with accumulation of microglia/macrophages in EAE [13], and it has been shown that CCR2 depletion reduced APP immunoreactivity after TBI [18]. Given that impairment of CCR2 signaling either by genetic deletion or by pharmacological intervention ameliorates TBI in rodents [26–29], we hypothesized that reduced monocytes infiltration might lead to less neuronal damage.

**Fig. 3:**
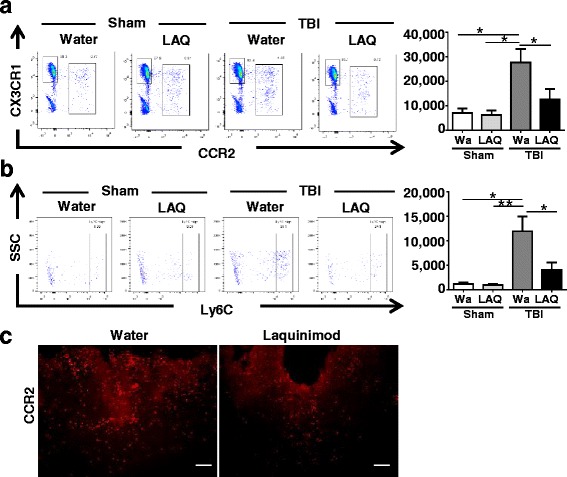
Laquinimod reduced monocytes infiltration: **a**–**b** Representative images of cortex at 3 days after TBI. **a**–**b** Flow cytometry analysis to look at infiltrated monocytes in the brain. **a** shows CD45 gated cells and total monocytes determined as CD45^+^CX3CR1^low^CCR2^+^ cells. **b** shows that CD45^+^CX3CR1^low^CCR2^+^ gated cells and Ly6C^high^ cells are infiltrating monocytes. Quantification of both total and infiltrating monocytes revealed increase in TBI-water group and reduction in TBI-laquinimod group. **c** Sections stained for RFP to visualize infiltrating peripherally derived monocytes. We studied five to seven mice per group from at least three independent experiments. Scale bar, 100 μm. Symbols denote significant difference between groups. Error bars indicate mean ± s.e.m. (*n* = 3–6/group). *p < 0.05, **p < 0.01

Flow cytometric analysis revealed that laquinimod treatment decreased total CCR2^+^ monocytes, as well as Ly6C^+^ monocytes in the brain, immune cells known to be recruited to the CNS following inflammatory insults (*p < 0.05) (Fig. 3a, b). Infiltrating monocytes in the brain were reduced by 66.2% (Average Ly6C^+^ cell number in the TBI-water group; 11,907/mouse, in the TBI-laquinimod group; 4027/mouse) in TBI-laquinimod group. Tissue staining for CCR2 showed that CCR2^+^ monocytes were mostly distributed around vessels in TBI-water group and not affected by laquinimod (Fig. 3c). These results suggest that the reduced numbers of CCR2^+^ cells in TBI-laquinimod tissues are not associated with a change in distribution of CCR2^+^ cells.

### Laquinimod suppressed inflammation-related genes on monocytes

**Fig. 4:**
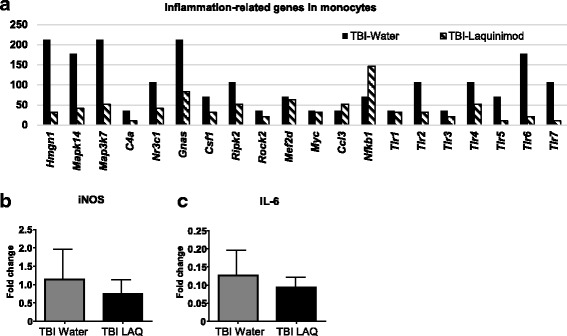
Inflammatory responses are suppressed by laquinimod in monocytes following TBI. **a** Gene expression of inflammatory-related molecules in peripherally derived monocytes as measured by MG468 chip. **b–c** qPCR validation of *iNOS* (**b**) and *IL-6* (**c**) in peripherally derived monocytes. We studied five to seven mice per group from at least three independent experiments. Bars show mean ± s.e.m. (*n* = 5)

To investigate the monocytes’ role on TBI in the acute phase of TBI, we sorted infiltrated monocytes at 3 days post injury and performed gene expression analysis using Nanostring system [19, 20]. Comparing gene expression changes of the laquinimod-treated TBI group with that of the water control TBI group, 24 genes were upregulated by laquinimod. On the other hand, more than half of the genes examined (259/468 genes) were downregulated by laquinimod. In addition, inflammation-related genes (*Tlr5*, *Hmgn1*, *Mapk14*, *Map3k7*, *Tlr2*, *C4a*, *Csf1*, *Pitpnm1*, *Nr3c1*, *Gnas*, and *Ripk2*) and *tlr3*, *tlr4*, *tlr7*, *and tlr7* were downregulated by laquinimod in monocytes (Fig. 4a).

Nitric oxide (NO) derived from the inducible isoform of NO synthase (iNOS) is an inflammatory product implicated in secondary damage from brain injury, and either iNOS-deficiency or iNOS inhibitors are protective at the early phase of injury [30–32]. Interleukin 6 (IL-6) is another potential mediator of TBI [33, 34] and high level of IL-6 in cerebrospinal fluid (CSF) or serum has reported as high risk for poor outcomes in human [35, 36]. To address whether iNOS or IL-6 production were affected by gene expression changes in monocytes, we performed quantitative RT-PCR. Quantitative RT-PCR showed a tendency of decrease in iNOS (34.4%) and IL-6 (26.3%) production in monocytes treated by laquinimod (Fig. 4b, c). Taken together the effects of laquinimod on inflammatory gene expression (about one-third reduction on a per-cell basis) and on monocyte infiltration (about two-thirds reduction in total infiltrated cells), we estimate that overall monocyte inflammatory gene expression in the CNS of laquinimod-treated mice was lowered by as much as 80%.

### TBI-laquinimod microglial gene expression resembles the sham control group more than TBI-water group

**Fig. 5:**
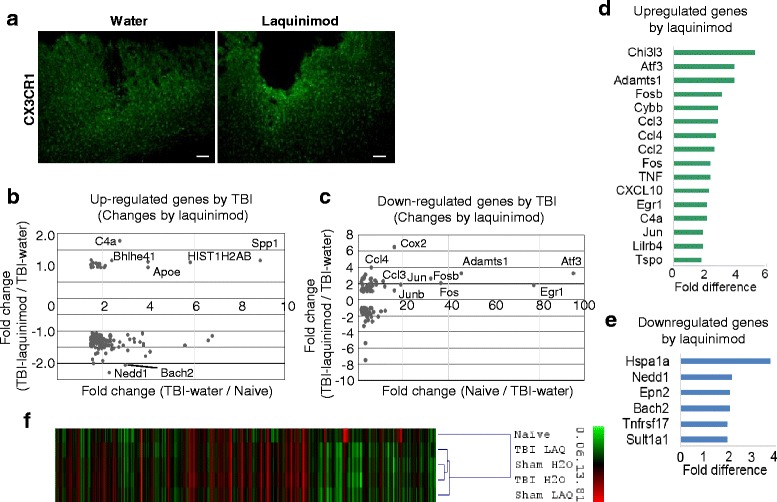
Laquinimod promote a more sham-like microglial phenotype: **a** Sections stained for GFP to visualize microglia. **b–c** Fold change of upregulated (**b**) or downregulated (**c**) microglia genes following TBI with or without laquinimod treatment. Note that transcription genes are upregulated with laquinimod. **d–e** Fold changes of upregulated (**d**) or downregulated (**e**) microglia genes of TBI-laquinimod group compared to TBI-water group. **f** Hierarchical clustering of nanostring data from naïve, sham, and TBI of different treatment condition shows that laquinimod treatment attenuated TBI-induced microglial gene expression closer to the sham group. We studied five to seven mice per group from at least three independent experiments. Scale bar, 100 μm. *p < 0.05, **p < 0.01

Next, we addressed how laquinimod might affect microglial functions in TBI pathology. The involvement of microglia in the pathophysiological cascade following brain injury has long been recognized. As expected, the injury induced microglial morphological transformation (ramified, bushy) with increased GFP signal at the site of damage in the TBI-water group (Fig. 5a). Similarly, TBI-laquinimod showed increased GFP signal under the damaged site and microglial distribution nor their morphology were not altered by the treatment (Fig. 5a).

Next, we sorted microglia and compared gene expression changes. From the comparison between mice with TBI and naïve mice group, we found that 124 genes expression were elevated in microglia by TBI. Conversely, there were 112 downregulated genes including transcriptional factors (*Atf3*, *Egr1*, *Fos*, *Fosb*, *Jun*, *Junb*), which are important for cell proliferation and differentiation.

Among 124 genes that increased their expression after TBI, most of them did not change their expression after laquinimod treatment (Fig. 5b). Five genes (Spp1, *HIST1H2AB*, *Apoe*, *C4a*, *and Bhlhe41*) slightly elevated their expression and two genes (*Bach* and *Nedd1*) decreased their expression. Among 112 genes that decreased their expression after TBI, six genes including transcription factors and macrophage inflammatory proteins (MIP)-1 chemokines (*Atf3*, *Adamts1*, *Fos*, *Fosb*, *Ccl3*, *Ccl4*) elevated their expression in response to laquinimod, although the expression level was still significantly lower than that of naïve group (Fig. 5c). Upregulated genes by laquinimod compared with the TBI-water group were listed in Fig. 5d. The level of *Chi3l3* (chintinase, Ym1), a target gene of IL-4 and STAT6 pathway and one of anti-inflammatory markers, was not altered by TBI and increased by laquinimod. As described above, there were transcriptional factors important for cell proliferation and differentiation (Atf3, Fosb, Fos, Egr1, Jun); however, their level were still considerably lower than that of naïve group. Six genes were downregulated by laquinimod compared with those in the TBI-water group (Fig. 5e).

Finally, hierarchical clustering analysis was performed on the genes from each group using the Multi Experiment Viewer (MeV) software. The gene expression cluster of the TBI-water group and the sham-water control group was the most similar among all the groups. Subsequently, the laquinimod-treated TBI and sham group showed the similarity to TBI-water group and the sham-water control group (Fig. 2b). It is noteworthy that the TBI-water group and the TBI-laquinimod group do not cluster particularly closely. These results indicate that laquinimod attenuated TBI-microglial gene expression closer to the sham group.

## Discussion

Activation of microglia and infiltration of peripherally derived monocytes are key responses to the injured brain after TBI. Since laquinimod has been reported to modify myeloid functions in other CNS inflammatory models, we wanted to examine if laquinimod treatment will also affect TBI-induced inflammation. Herein, we provided the effectiveness of laquinimod for TBI mouse model: First, administration of laquinimod was able to prevent the brain atrophy in the long term, which may have contributed by preserved axonal integrity and restored hippocampal neurogenesis. Secondly, laquinimod significantly reduced monocyte infiltration to the CNS. Finally, laquinimod promoted microglial phenotype close to the sham control group rather than TBI-water group.

Functions of laquinimod are diverse. In EAE model, it has been reported that laquinimod reduced infiltration of immune cells into the CNS and suppressed inflammatory T cell responses [37, 38]. Apart from the effects on myeloid cells, reduction of the lymphocyte adhesiveness to VCAM-1 [38], reduced expression of CCL2, the molecule that regulates the trafficking of monocytes [12], and decreased chemokine production by dendritic cells [39] with laquinimod treatment have also been reported. In vitro experiments also showed that laquinimod reduced the activity of pathways including Jun-N-terminal kinase in LPS-activated microglia [13].

We took advantage of using *Ccr2*^*rfp/+*^ *Cx3cr1*^*gfp/+*^ mice to distinguish microglia and monocytes in the CNS. We recently described that *Ccr2* deletion, but not *Cx3cr1* deletion, reduced cavity volume and axonal pathology by inhibiting monocyte infiltration at an early time point (3 days post-injury) following TBI [18]. In the current study, we found that infiltration of CCR2^+^ monocytes after TBI was significantly and substantially reduced by laquinimod treatment and that treatment with laquinimod also leads to similar neuroprotective effects. For instance, the beneficial effects of laquinimod treatment on neurons were evident by the finding that in treated mice, most of the neurons showed very low or complete lack of the axonal damage marker, APP, expression whereas in untreated mice, the vast majority of the neurons were positive for APP. Additionally, laquinimod treatment also restored hippocampal neurogenesis.

The mechanisms of protection remains incompletely defined. Given that impairment of CCR2 signaling ameliorates TBI in rodents, we propose the hypothesis that reduced monocyte infiltration underlies some or all of the benefits we observed. As CCR2 is a chemokine receptor, mostly expressed by peripheral monocytes, which orchestrates the recruitment of these immune cells to the CNS, it is quite reasonable that inhibiting monocyte migration either through Ccr2 modification or laquinimod administration might be a key factor for neuronal rescue following TBI. Interestingly, the majority of the inflammation-related genes evaluated were downregulated by laquinimod in infiltrated monocytes; however, the reduction was not so significant in microglia (Additional file 1). Tendency of reduced iNOS and IL-6 gene expression in laquinimod-treated monocytes possibly play a role in decreased axonal damage (Fig. 2) [40]. Additionally, the laquinimod downregulated the expression of most of TLRs (TLR2, 4, 5, 6, and 7) and p38MAPK (*Mapk14*), but not c-JUN or NFκB, in monocytes (Fig. 4). The TLR signaling pathway activates NFκB, resulting in the upregulation of many inflammatory genes (e.g., cytokines, chemokines, COX-2, and iNOS) [41]. It is possible that laquinimod also attenuates the activation of inflammatory proteins by minimizing and/or preventing TLR signaling, leading to inhibition of inflammatory p38MAPK pathway. However, additional studies are necessary to better clarify this issue.

The microglial response in TBI is dualistic and highly dependent upon timing and nature of the injury [42–44]. While CCR2 deficiency impairs monocytes infiltration and improves functional recovery and neuronal survival (Gyoneva et al. [18]; Hsieh et al. [27]), CX3CR1 deficiency has showed a time-dependent effect after TBI, i.e., early protection but late worsening [43, 44]. Herein, transcription factors that promote “healthy” microglial phenotype were significantly downregulated following TBI. Importantly, laquinimod treatment did not suppress these transcript factors, being favorable to healthy microglial phenotype maintenance, which might explain, at least in part, laquinimod neuroprotective effect.

Hierarchical clustering provided insights into gene expression patterns of several conditions of microglia. Naïve pattern was totally different from sham and TBI groups. Microglia from sham-water group showed a similar pattern to that from TBI-water group rather than injured side of TBI-laquinimod group, which suggests that laquinimod succeeded in modifying gene expression in TBI towards sham-like gene pattern.

There is emerging evidence that increased neuroinflammation leading to neurodegeneration is relevant for memory impairment as a result of brain injury caused by trauma [45], athletic sports [46], or military [47]. Chronic traumatic encephalopathy (CTE) has gained attention, which is seen in people exposed to repetitive head injury [24]. Neuropathological features of CTE include brain atrophy, ventricular enlargement, amyloid-β and tau, and TDP-43 pathologies, many of which might contribute to cognitive decline [24, 48]. Similar pathological changes are also commonly found years after a single moderate to severe TBI. Laquinimod treatment at TBI acute phase led to histological improvement in ventriculomegaly at a later time point, 120-days post-injury, indicating its potential to prevent long-term TBI-related neuropathological changes.

Apart from the robust findings, our study has some limitations. Preliminary gene expression studies, using nCounter technology and MG468 array, were performed in separately sorted microglia and monocytes to determine whether further insight into the effects of laquinimod on myeloid cells might clarify the beneficial effects of this treatment. The results were inconclusive and are provided to the scientific community to increase the data base of information related to response of myeloid cells to TBI and to laquinimod.

We focused our experiments on day 3 post injury, when the number of infiltrating monocytes is at a peak, in order to demonstrate and characterize potential differences in the response of infiltrating monocytes and resident microglia to a relevant disease model at a pre-specified time point. On the other hand, laquinimod shows its effect 3 to 4 days after the administration in EAE [49]. For this reason, we conducted a pretreatment in order to guarantee laquinimod effects. Further studies need to address the effect of administration of laquinimod after TBI. Regarding behavioral and cognitive assessment, there were no cognitive deficits in the TBI group compared to the sham group, and therefore, we were not able to detect the role of laquinimod on behavioral function (data not shown). One possibility for this is that the injury was not strong enough to lead behavioral and cognitive abnormalities. However, despite the lack of functional changes, we provided evidence that laquinimod present a neuroprotective role in acute TBI and also prevent the long-term enlargement of lateral ventricle (Fig. 1). More severe injuries may be required to emphasize the difference.

In summary, we report a pronounced benefit of laquinimod treatment for lateral fluid percussion TBI in adult mice. Importantly, laquinimod seems to confer neuroprotection following TBI by distinctly modulating microglia and monocyte functions and microglia to promote anti-inflammatory responses, while modulates monocyte infiltration and inhibits their inflammation-related gene expression. It is worth noticing that laquinimod does not neutralize nor contradict the function of cells. The current study brought new insights into molecular and cellular mechanisms underlying TBI pathophysiology, paving also a road for the study of laquinimod as a promising therapeutic strategy.

## Conclusions

TBI is a major risk factor for the development of neurodegenerative diseases. The development of effective therapeutic strategies is a scientific goal of highest priority in order to prevent secondary damage to primarily unaffected tissues. Our data indicated for the first time multifaceted roles of laquinimod, including immunomodulatory and neuroprotective mechanisms of action in response to TBI. Laquinimod is an immunomodulatory drug used in the clinical practice for the treatment of MS. Our findings provide preclinical evidence of therapeutic benefit of laquinimod in TBI.

## Additional files

Additional file 1: Inflammation-related gene expression changes in microglia. A, Gene expression of inflammatory-related molecules in microglia as measured by MG468 chip. B–C, qPCR validation of *iNOS* (B) and *IL-6* (C) in microglia. We studied 5–7 mice per group from at least three independent experiments. Bars show mean ± s.e.m. (*n* = 5). (PDF 97 kb)

Additional file 2: Gene data—TBI and laquinimod. Sheet 1: nCounter CodeSet Design. Sheet 2: MG468 Isoform Coverage. Sheet 3: MG468 Annotated genes. Sheet 4: Normalized data. Sheet 5: List of all detected genes. (XLSX 953 kb)

## Abbreviations

APP: Amyloid precursor protein
CCR2: Chemokine (C-C motif) receptor 2
CNS: Central nervous system
CX3CR1: Chemokine (C-X3-C motif) receptor 1
GFP: Green fluorescent protein
LFPI: Lateral fluid percussion injury
NGS: Normal goat serum
PBS: Phosphate-buffered saline
RFP: Red fluorescent protein
TBI: Traumatic brain injury
