# Supplementary figures and images for: Laquinimod attenuates inflammation by modulating macrophage functions in traumatic brain injury mouse model
Source: J Neuroinflammation. 2018 Jan 30;15:26. doi: 10.1186/s12974-018-1075-y (PMC5791334; doi:10.1186/s12974-018-1075-y)

A

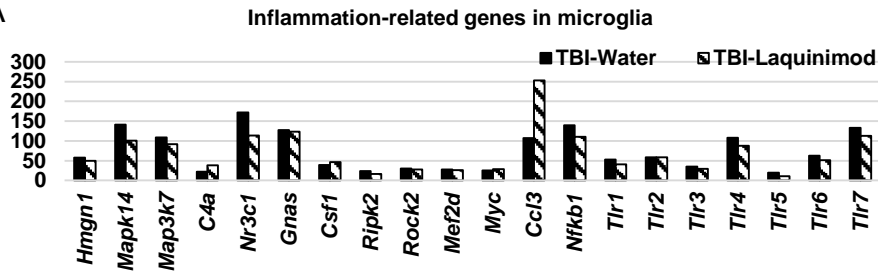

B

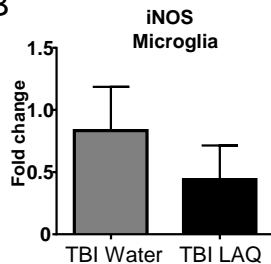

C

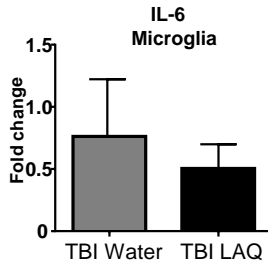

Supplement: Supplementary file 1 — Inflammation-related gene expression changes in microglia. A, Gene expression of inflammatory-related molecules in microglia as measured by MG468 chip. B–C, qPCR validation of iNOS (B) and IL-6 (C) in microglia. We studied 5–7 mice per group from at least three independent experiments. Bars show mean ± s.e.m. (n = 5). (PDF 97 kb) [file 12974_2018_1075_MOESM1_ESM.pdf]
